# Case report: Self-restraint in a patient with alien hand syndrome following cerebral infarction involving the anterior cerebral artery territory

**DOI:** 10.3389/fneur.2023.1203450

**Published:** 2023-07-14

**Authors:** Kazuhiro Sugawara, Toshiki Takeuchi, Kuniaki Harada, Marina Taki, Ikumi Fujimura, Yuichi Kogami, Ryoichi Furuta

**Affiliations:** ^1^Department of Physical Therapy, School of Health Sciences, Sapporo Medical University, Sapporo, Japan; ^2^Department of Rehabilitation, Kashiwaba Neurosurgical Hospital, Sapporo, Japan; ^3^Graduate School of Health Sciences, Sapporo Medical University, Sapporo, Japan; ^4^Department of Radiological Sciences, Faculty of Health Sciences, Japan Healthcare University, Sapporo, Japan

**Keywords:** cerebral infarction, corpus callosum, alien hand syndrome, diffusion tensor imaging, self-restraint

## Abstract

Frontal alien hand syndrome (AHS) presents as impulsive grasping and groping and compulsive manipulation of environmental objects that can affect the dominant or nondominant hand. A few reports have shown improvements in neuropsychological scores over time when self-restraint of the right hand AHS was enforced. A 72-year-old woman presented with right-handed involuntary instinctive grasping reactions and compulsive manipulation of tools after an infarction of the frontal lobe and corpus callosum (CC). She was diagnosed with cerebral infarction involving the anterior cerebral artery territory and a frontal variant of AHS. At AHS onset, the patient was unaware that her right hand was moving against her will; she was only aware that her right hand was moving when the therapist pointed it out to her. Later, she began to recognize that her right hand was involuntarily moving, and she could restrain the movement of her right hand with her left hand. Approximately 5 months following AHS onset, the patient could voluntarily restrain her AHS symptoms by telling her right hand not to move against her will in her head. Most neuropsychological scores improved by 5 months following AHS onset. However, the patient showed disruptions in the genu and midbody of the left cingulate cortex, as shown via diffusion tensor imaging (DTI), and the sensation of the “right hand moving by itself” remained even 5 months after AHS onset. Although damage to the CC fibers was evident on DTI at 5 months following onset, the patient exhibited no sensory deficits and demonstrated good hand ownership as well as early improvement in attention and cognitive dysfunction. Therefore, the patient recognized her AHS symptoms, which included her hand moving against her will, and was able to consciously restrain her hand movement.

## 1. Introduction

Alien hand syndrome (AHS) is characterized by the involuntary and autonomous activity of the affected limbs and includes frontal, callosal, and posterior variants (1, 2). Frontal AHS presents with impulsive grasping and groping and compulsive manipulation of environmental objects in the presence of frontal-release signs and can affect the dominant or nondominant hand (3). Although the long-term course of frontal AHS symptoms has been described, to the best of our knowledge, the disease course including the results of neuropsychological tests has not been reported, and no cases of patients who were able to self-restrain abnormal movement related to AHS symptoms have been reported.

Herein, we describe a case of right-handed AHS after cerebral infarction in the left anterior cerebral artery region whose symptoms improved neuropsychologically over time and enabled the self-restraint of right-handed AHS. Diffusion tensor imaging (DTI) of the patient is shown and the basis of fiber contacts in the corpus callosum (CC) at 5 months after onset is discussed. The following case is presented in accordance with the CARE reporting checklist.

## 2. Case presentation

A 72-year-old right-handed woman was admitted to our hospital with a history of hypertension and diabetes mellitus (DM). The patient had been diagnosed with hypertension and diabetes 2 years before the current hospital admission and attended a specialized clinic where her condition was controlled using medications for the respective illnesses. The patient took Valsartan 80 mg for hypertension and Glimepiride 1 mg for DM. She had no other medication history or relevant family history. Informed written consent for the publication of the clinical details of this case was obtained from the patient. After falling at the entrance of her house, the patient could not walk and had required external support to walk. The patient was admitted to our hospital's neurology department owing to poor response and difficulty in standing the next day. She was diagnosed with cerebral infarction involving the anterior cerebral artery territory and a frontal variant of AHS. On admission, hematological tests including a complete blood count and coagulation fibrinolysis examination revealed no obvious abnormalities (white blood corpuscle, 4.7 × 10^3^/μl; red blood corpuscle, 461 × 10^4^/μl; hemoglobin, 14.1 g/dl; hematocrit, 41.8%; platelet count, 10.9 × 10^4^/μl; fibrinogen, 296 mg/dl; and triglycerides, 70 mg/dl), with items related to blood glucose levels in biochemical tests alone being elevated (albumin, 4.2 g/dl; glucose, 124 mg/dl; and hemoglobin A1c, 6.4%). The patient was conscious and had Brunnstrom stage V in her right upper and lower limbs. The Romberg test was negative. The Babinski and Chaddock signs in her right lower extremity and the Hoffmann reflex in her right upper extremity were negative. She had mild right facial paralysis and was alert and oriented with nonfluent speech and slight dysarthria. The palate was asymmetrical on phonation, and the tongue was slightly shifted to the left. Coordination was slightly worse on the right upper extremity, as assessed using the finger–nose–finger test and pronation–supination test. Superficial and deep sensations were normal. No diagnostic dyspraxia was observed. The patient was administered aspirin antiplatelet therapy (100 mg QD) for the treatment of her cerebral infarction. She could not release another person's arm or object after grasping them once with her right hand and pressed all the buttons of the elevator and the nurse call with her right hand involuntarily. The patient's Hasegawa's Dementia Scale-Revised (HDS-R) score was 11, Mini-Mental State Examination (MMSE) score was 17, Frontal Assessment Battery (FAB) score was 5, Praxis as one subscale in the Western Aphasia Battery (WAB) score was 43 for the right hand and 35 for the left hand (Table 1), and there was no apraxia in her daily life.

**Table 1:** Changes in the scores of the neuropsychological tests over time from admission to discharge.

	**Admission**	**1 month**	**2 months**	**3 months**	**4 months**	**5 month (discharge)**
HDS-R (/30)	11	18	22	26	28	28
MMSE (/30)	17	22	26	28	28	30
FAB (/18)	5	10	11	16	15	13
WAB (right) (/60)	43	60	58	59	60	60
WAB (left) (/60)	35	58	58	60	60	60
TMT-part A (sec) (errors)		147 (2)	139 (0)	77 (0)	79 (0)	87 (0)
TMT-part B (sec) (errors)		–	316 (5)	235 (2)	181 (3)	183 (4)
BIT-total (/146)		132	141	142	144	139
Kohs block-design test (IQ)				64	64	57.8

HDS-R, Hasegawa's Dementia Scale-Revised; MMSE, Mini Mental State Examination; FAB, Frontal Assessment Battery; WAB, Praxis as one subscale in Western Aphasia Battery; BIT-total, Behavioral Inattention Test.

**Figure 1:**
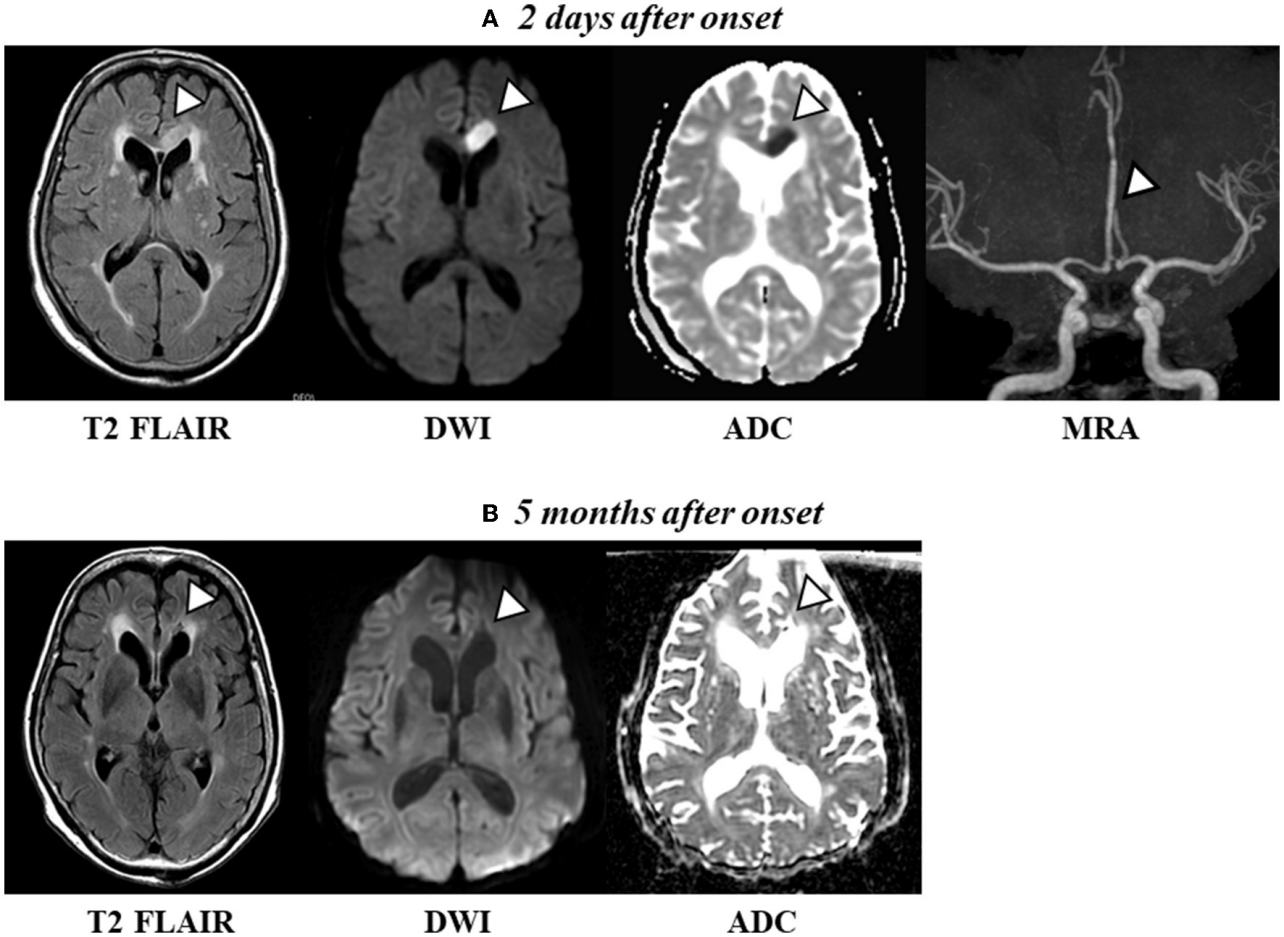
**(A)** Axial T2 FLAIR scan and diffusion-weighted imaging (DWI) revealed a high-intensity signal [apparent diffusion coefficient (ADC) shows a low signal intensity, indicating acute cerebral ischemia] in the left frontal lobe and midbody of the CC 2 days after onset. Magnetic resonance angiography (MRA) images revealed a vessel occlusion of the left A2. **(B)** Axial T2 FLAIR and DWI show a change to a low-intensity signal (conversely, ADC shows a high-intensity signal, indicating a chronic phase) in the left frontal lobe and the midbody of the CC 5 months after onset.

Magnetic resonance imaging (MRI) of the brain 2 days after AHS onset revealed a high-intensity signal in the left anterior cerebral artery region (the left genu to midbody of CC) on admission (Figure 1A), and a low-intensity signal in the same regions 5 months following onset (Figure 1B). Computed tomography and MRI did not reveal any hemorrhages or exudates. Magnetic resonance angiography (MRA) images revealed a vessel occlusion of the left A2 (Figure 1A). The vital signs of the patient were as follows: pulse, 52/min and blood pressure, 163/96 mm Hg. Her heart rate and rhythm were regular. Echocardiography revealed mild aortic, mitral, and tricuspid regurgitation but no organic change. No left ventricular hypertrophy or primary pulmonary hypertension was observed, and left ventricular contraction was normal. A transcranial Doppler study revealed a normal direction of blood flow with mean flow velocities and spectral waveform within normal limits in all insolated segments of the circle of Willis. In addition, DTI was performed at 5 months after onset.

As the patient had mild motor paralysis in the upper and lower limbs from AHS onset, therapy included walking and exercise of daily living to help the patient live independently in the ward. The patient was able to stand and walk without physical assistance. The patient underwent physical and occupational therapy for the upper limbs, focusing on bimanual movements and object manipulation exercises to treat her AHS. When compulsive manipulation of tool use and/or instinctive grasping reactions appeared, the therapist provided verbal instructions, and the patient shifted to verbal statements by herself over time.

A month after onset, score improvements were observed in the HDS-R, MMSE, and FAB and score reduction was observed in the BIT-total in “Figure and shape copying” and “Representational drawing.” The Trail Making Test (TMT)-part A took 147 s and she made two errors; the TMT-part B could not be completed (Table 1). The patient's instinctive grasping reaction to the handrail remained involuntary, and she also continued to repeatedly press the nurse call button and push the elevator buttons. Furthermore, she wrote her name with her right hand against her intention when a pen was placed in front of her. However, the abnormal movement of the right hand could be corrected by verbal instructions from the therapist or other interventionists.

Three months following AHS onset, compared with a month after onset, there were further improvements in the scores of the HDS-R, MMSE, and FAB as well as improvements in performance in the TMT-A and -B. At this time, there was no apraxia on the WAB, and performance on the Kohs block-design test was low (Table 1). The patient was able to restrain her right hand with her left hand, although she still repeatedly pressed the nurse call button and the elevator buttons.

Five months after AHS onset, the patient made a statement that “the right hand moves on its own,” but “I can prevent the right hand from moving in my head before it starts to move,” and the number of right hand movements against her intention decreased. The scores of the FAB, TMT-A and -B, BIT-total, and Kohs block-design test decreased (Table 1); however, these tests were conducted just before discharge; thus, it was possible that her mental agitation affected these tests.

MRI was performed using a 3.0 Tesla MRI scanner (Discovery MR750; GE Healthcare, Milwaukee, Wisconsin). The patient was scanned in the supine position using a GEM Head & Neck coil. DTI was performed using a special sensitivity array encoding protocol, with factor = 2, chemical shift-selective suppression, and an echo-planar imaging sequence. An image without diffusion encoding (*b* value = 0 s/mm^2^) was acquired to register the diffusion-weighted volume during analysis. The following imaging parameters were used: *b* value = 1,000 s/mm^2^, motion probing gradient, 15 directions, time to repetition/echo time = 8,500/109.6 ms, flip angle = 90°, axial slice orientation, slice thickness = 3.6 mm with no interslice gap, field of view = 260 × 260 mm, 128 × 128 matrices, 40 slices, number of acquisitions = 2, and a 4-min and 40-s scan time. The regions of interest were manually selected separately on sagittal fractional anisotropy colormaps for each of the bilateral genu, midbody, and splenium of the CC, and the fractional anisotropy (FA) values were calculated as the mean of the individual values obtained from each region of interest.

**Figure 2:**
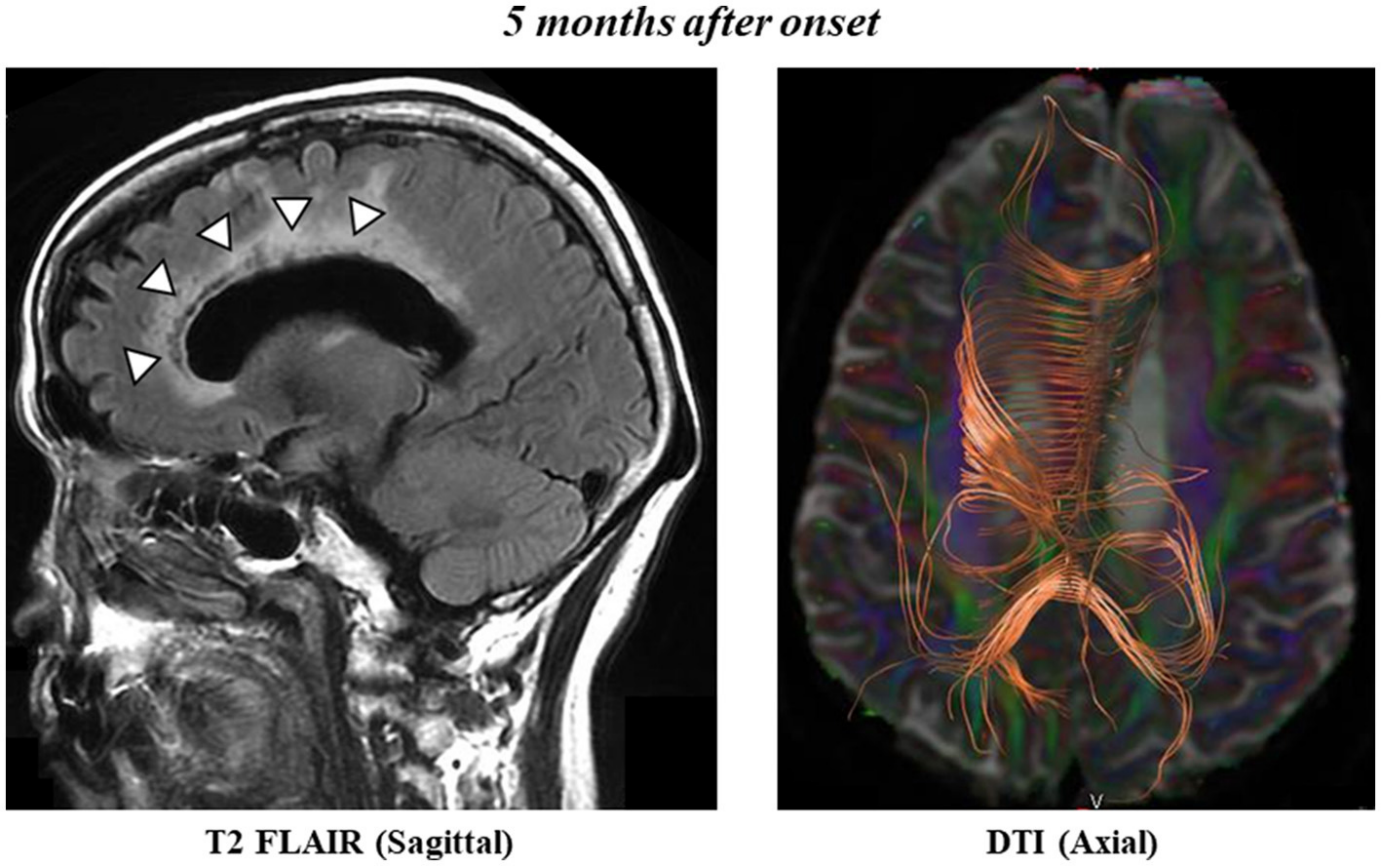
Sagittal T2 FLAIR scan shows postinfarct necrosis in the left frontal lobe, genu, and midbody of the CC 5 months after onset **(left panel)**. Axial diffusion tensor tractography of the CC 5 months after onset **(right panel)**.

MRI T2-FLAIR revealed low-intensity signals and enlarged left cerebral ventricles 5 months after onset (Figure 2, left panel). DTI showed disruptions in the genu and midbody of the left CC (Figure 2, right panel), which were bilaterally connected to the frontal and parietal cortices, respectively. We also observed CC fibers in the splenium of the CC. The FA values were 0.64 for the genu of the right CC, 0.29 for the genu of the left CC, 0.67 for the midbody of the right CC, and 0.46 for the midbody of the left CC, consistent with the values of the infarcted region. The FA values in the splenium of the CC were 0.80 and 0.72. These results confirm that the cerebral CC fibers had not recovered even after 5 months.

## 3. Discussion

We presented the case of a patient with cerebral infarction in the left anterior cerebral artery region who presented with AHS due to damage from the left genu to midbody of the CC. Although motor paralysis and sensory impairment were very mild, AHS symptoms, including instinctive grasping reaction and compulsive manipulation of tools, were observed. With time, the patient was able to restrain the unintended movements of her right hand.

Feinberg et al. (3) reported compulsive tool use as a characteristic symptom of frontal AHS. Frontal AHS lesions are usually located in the medial frontal lobe (supplementary motor area, anterior cingulate gyrus, and medial prefrontal cortex) and the anterior part of the CC, including the genu (4–6). In this case, MRI revealed an ischemic lesion in the left supplementary motor and medial frontal cortices. In addition, the patient's compulsive manipulation of tools, such as repeatedly pushing the nurse call and elevator buttons regardless of the patient's intention, was confirmed, suggesting a high probability that the patient had frontal AHS. Kikkert et al. (6) reported that AHS symptoms decreased in 68% of patients within 1 year of onset. Herein, the postinfarct necrosis of the genu and midbody of the CC was confirmed 5 months after onset via MRI, and the patient still had the sensation of the “right hand moving by itself” at the time of hospital discharge, suggesting that AHS was still present 5 months after onset.

DTI data at 5 months revealed that the left genu and midbody of CC were disconnected from the left and right sides of the hemispheres, consistent with the data of the infarcted region, resulting in lower FA values. FA values represent the degree of directionality of microstructures, such as axons, myelin, and microtubules, and the tract number is determined by counting the number of voxels contained within a neural tract (7, 8). Herein, the connection in the genu and midbody of the CC did not recover, indicating that AHS had not resolved. Therefore, the DTI data in this case strongly support the introspection of the “right hand moving by itself” at 5 months after onset.

The patient restrained AHS over time, although she still exhibited AHS symptoms. At onset, the patient was unaware that her right hand was moving against her will; she was only aware that her right hand was moving when the therapist pointed it out to her. Later, she began to recognize that her right hand was moving without her intention, and she could restrain the movement of her right hand with her left hand. Approximately 5 months after onset, the patient was able to voluntarily restrain the AHS symptoms by telling her right hand not to move against her will in her head. The patient exhibited little motor paralysis and sensory impairment compared with that at onset, and the sense of her right-hand ownership remained. The scores of the neuropsychological tests improved early during the disease course; however, the AHS symptoms persisted until the patient was discharged from the hospital, although the patient was able to control herself using her left hand (contralateral to the upper limb where symptoms appeared) or via her own will.

## 4. Conclusion

Although damage to the CC fibers was evident on DTI at 5 months after onset, the patient exhibited no sensory deficits and showed good hand ownership as well as early improvements in attention and cognitive dysfunction. Therefore, the patient could recognize the AHS symptoms (hand movement against her own will) and was able to consciously restrain this movement. However, the direct causal relationship between the scores of the neuropsychological tests and AHS remains unclear. Further investigation focused on the relationships between cognitive functions and AHS prognosis is warranted.

## Data availability statement

The raw data supporting the conclusions of this article will be made available by the authors, without undue reservation.

## Ethics statement

Written informed consent was obtained from the individual(s), and minor(s)' legal guardian/next of kin, for the publication of any potentially identifiable images or data included in this article.

## Author contributions

KS performed manuscript writing. KS, TT, YK, and RF performed literature reviews. TT, MT, and IF were involved in patient care. KH conducted and operated the MRI. All authors approved the final version of the manuscript.
